# The Incidence and Risk Factors for Lower Limb Skin Graft Failure

**DOI:** 10.1155/2014/582080

**Published:** 2014-07-15

**Authors:** Sumeet Reddy, Falah El-Haddawi, Michael Fancourt, Glenn Farrant, William Gilkison, Nigel Henderson, Stephen Kyle, Damien Mosquera

**Affiliations:** Department of General Surgery, Taranaki Base Hospital, Private Bag 2016, New Plymouth 4342, New Zealand

## Abstract

Lower limb skin grafts are thought to have higher failure rates than skin grafts in other sites of the body. Currently, there is a paucity of literature on specific factors associated with lower limb skin graft failure. We present a series of 70 lower limb skin grafts in 50 patients with outcomes at 6 weeks. One-third of lower limb skin grafts went on to fail with increased BMI, peripheral vascular disease, and immunosuppressant medication use identified as significant risk factors.

## 1. Introduction 

The use of skin grafts to aid in the healing of wounds was first described by the ancient Indians over 2,500 years ago [1]. Although operative techniques have evolved over time, the principles of successful grafting have remained the same. Intrinsic and extrinsic factors unique to each patient can be the difference between success and failure [2]. This is especially apparent in the lower limb, where skin grafts have higher failure and complication rates than in other areas of the body [3, 4]. Currently, there is a paucity of research focused on factors contributing to lower limb skin graft failure and this may in part explain the heterogeneity with which clinicians manage patients requiring lower limb skin grafts [5]. The aim of this study was to determine the incidence of failure of lower limb skin grafts and to identify contributing factors.

## 2. Methods

A prospective observational study of all consecutive patients requiring lower limb skin grafts operated on between December 2012 and December 2013 was undertaken. Skin grafts were performed using well-established techniques. All operations were performed under general or regional anaesthetic with prophylactic antibiotics. Split thickness skin grafts (STSG) were harvested using an air dermatome (Zimmer, Warsaw, IN, USA) and full thickness grafts (FTSG) were harvested using a scalpel with subcutaneous tissue removed prior to application. STSG were typically meshed prior to application and grafts were fixed with sutures, staples, or Dermabond (Johnson & Johnson, Ethicon Inc., Somerville, NJ, USA). Cuticerin (Smith & Nephew, London, UK) was applied over the graft with either a standard sponge bolster or negative pressure dressing (PICO TM, Smith & Nephew, London, UK). Patients were then either admitted to hospital for a 3–7-day period of bed rest with low molecular weight heparin or discharged with immediate mobilisation at the discretion of the surgeon. Grafts were reviewed at 2 and 6 weeks postoperatively. A skin was deemed successful if greater than 80% graft take has occurred on clinical examination. Data was entered into Microsoft Excel (Microsoft Corp., Redmond, WA, USA). Statistical analysis was done with SPSS 21 (Chicago, IL, USA). Normal distribution for statistical analysis was assumed with a parametric *t*-test and fisher's exact univariate analysis was used to determine significance.

## 3. Results

In total, 70 skin grafts were performed on 51 patients; 14 patients had multiple grafts performed. Baseline demographic and comorbidity data is shown in Table 1, the median age of the participants was 79 (range: 56–94 years old), and the majority of patients were female (57%, *n* = 29). The median BMI was 30 (range: 20−69), and nearly half of the patients had venous insufficiency and ischemic heart disease. There were also a high proportion of patients on immunosuppressant medication (8%, *n* = 4), and 11 patients (22%) had diabetes and peripheral vascular disease (PVD).

Elective surgery was performed in the vast majority of grafts (Table 2) and the main indication for surgery was skin cancer treatment. Over 2/3 of the grafts had placement of negative pressure dressing and placed on bed rest. The overall success rates of the grafts were 94%, 76%, and 67% at first inspection, 2 weeks, and 6 weeks, respectively. 17 grafts (24%) developed infection requiring antibiotics and 6 grafts (9%) developed a hematoma or seroma.

Bed rest and negative pressure dressing did not appear to be associated with increased graft success. The factors associated with graft failure were PVD, increased BMI, and use of immunosuppressant medications (Table 3). All failed skin grafts have gone onto to heal by secondary intention and no patients have required revision skin grafting procedures.

## 4. Discussion

In our experience, one-third of lower limb skin grafts failed at 6 weeks. Literature has reported rates of failure in lower limbs grafts of between 0 and 33% [6]. However, these rates are in a heterogeneous population with a variety of different indications, operative techniques, and followup. In addition to PVD and immunosuppressant use, we found increased BMI to be strongly associated with skin graft failure. The association of increased BMI and skin graft failure has not been described before. Penington and Morrison had identified waist to hip ratio to be associated with FTSG failure in the head and neck region in 14 patients [7]. Obese individuals are at increased risk of wound complications including wound infection, dehiscence, hematoma, and seroma formation [8]. Local and cellular factors including reduced microperfusion and decreased tissue oxygenation have been thought to play a part in this [7, 8]. Studies to explore specific mechanisms and impact of obesity as independent risk factor for poor operative outcome are still a much needed area for future research.

In our study, there was no difference in graft success rates between STSG and FTSG. To our knowledge, no study has directly compared outcomes between STSG and FTSG in the lower limb. A prospective study randomised 68 patients undergoing elective operations requiring radial forearm free flaps into receiving STSG or FTSG to the radial forearm free flap donor site [9]. No difference in outcomes was seen between the two groups, although patients with STSG required significantly more wound dressing changes compared to those who had FTSG. FTSG are thought to be superior to STSG in terms of cosmesis and decreased donor site complications [1]. However, STSG remain the most common method of skin coverage in grafting of the lower limbs owing to better scar quality than healing by secondary intention, ease of use, and ability to expand coverage through meshing [10]. The wound defects in the lower limb are often too large to be closed primarily and local flap repair can be difficult to achieve especially in elderly populations. It is also simpler to undertake revision surgery and oncological surveillance in patients who have had skin graft repairs compared to those with local flap repairs [10].

No difference in outcomes or complications was seen between patients placed on bed rest and those immediately mobilised. The vast majority of patients requiring lower limb grafts were placed on bed rest by the operating surgeon in our study. Bed rest is still widely used throughout the world despite an increasing body of evidence showing no significant benefit in outcomes [11]. Its popularity may be partly due to the clinical observation of decreased tissue oedema and perceived less graft disruption with limb elevation and bed rest, especially in this population with high rates of venous insufficiency. Similarly, no benefit in graft success rate was seen with the use of negative pressure dressings; a recent Cochrane review found no evidence to support or refute the effectiveness of commercial negative pressure dressing to improve healing rates of skin grafts [12].

## 5. Conclusion

Lower limb skin grafts have high failure rates. Increased BMI, immunosuppressant use, and PVD appear to be significant risk factors associated with graft failure. Knowledge of these factors is important in preoperative assessment to identify patients at increased risk of postoperative complications. A larger prospective trial assessing the comparative effectiveness of different strategies aimed at minimising complications of lower limbs is needed.

## Figures and Tables

**Table 1:** Baseline factors of patients having lower limb skin grafts, data presented as (*n*, %) unless otherwise stated.

	*n* = 51
Age: median (range)	79 years old (56–94 years old)
Patients having multiple grafts	14 (28%)
Sex (male: female)	22 : 29
ASA: median	2.5
BMI: median (range)	30 (20–69)
Venous insufficiency	25 (49%)
Ischemic heart disease	25 (49%)
Diabetes	11 (22%)
Peripheral vascular disease	11 (22%)
Smoking	9 (18%)
Continued on anticoagulation/antiplatelet agent	10 (20%)
Immunosuppressant medication	4 (8%)

**Table 2:** Operative details of lower limb skin grafts, data presented as (*n*, %) unless otherwise stated.

	*n* = 70
Indication	
(i) Cancer	60 (86%)
(ii) Trauma	8 (11%)
(iii) Ulcer	2 (3%)
Elective case	59 (84%)
Surface area of graft: median (range)	0. 98 cm^2^ (0.12–8.8 cm^2^)
Type of graft	
(i) Split thickness	64 (91%)
(ii) Full thickness	6 (9%)
Type of dressing	
(i) Vacuum	49 (70%)
(ii) Sponge	21 (30%)
Management	
(i) Bed rest	48 (69%)
(ii) Immediate mobilization	22 (31%)

**Table 3:** Analysis of success grafts versus failed grafts, data presented as (*n*, %) unless otherwise stated.

	Graft success (*n* = 48)	Failure (*n* = 22)	*P* value
Age (median)	79 years old	78 years old	0.908
Sex (male : female)	21 : 27	8 : 14	0.753
Venous insufficiency	25 (52%)	15 (60%)	0.547
Ischemic heart disease	24 (50%)	13 (59%)	0.702
Diabetes	11 (23%)	8 (36%)	0.374
Peripheral vascular disease	20 (42%)	16 (73%)	0.030
Smoking	7 (15%)	5 (23%)	0.605
BMI (median)	30	42	0.007
Bed rest	32 (67%)	16 (73%)	0.829
Vacuum dressing	30 (63%)	19 (86%)	0.093
Split thickness skin graft	44 (92%)	20 (91%)	0.999
Immunosuppressants	1 (2%)	5 (22%)	0.020
Acute operations	7 (14.5%)	4 (18%)	0.951
Graft size (median)	0.94 cm^2^	1.28 cm^2^	0.331
